# A Comparative Evaluation of Time-dependent Changes on the Surface Hardness of Bulk Cure Composites: An *in vitro* Study

**DOI:** 10.5005/jp-journals-10005-1508

**Published:** 2018-06-01

**Authors:** Anindita Sarma, Priya Nagar

**Affiliations:** 1 Postgraduate Student, Department of Pedodontics and Preventive Dentistry Krishnadevaraya College of Dental Sciences & Hospital Bengaluru, Karnataka, India; 2 Head, Department of Pedodontics and Preventive Dentistry Krishnadevaraya College of Dental Sciences & Hospital Bengaluru, Karnataka, India

**Keywords:** Bulk cure composite, Flowable bulk cure composite, Vicker’s hardness.

## Abstract

**Objectives:**

The objective of this *in vitro* study was to assess the surface hardness through Vickers hardness (VH) test of one conventional hybrid resin composites (Filtek Z350), compared with that of two bulk cure resin composites (SDR™, Tetric N Ceram®).

**Materials and methods:**

Twenty specimens of each material were prepared in cylindrical aluminum molds with an internal diameter of 5 mm and depth of 4 mm, 10 (incremental curing) and 10 (bulk curing).

The surface of each specimen was covered with a transparent plastic matrix strip before light curing with conventional visible light for 40 seconds. The specimens thus obtained were stored in deionized water and transferred to an incubator at 37°C for 24 hours to simulate clinical conditions. After 24 hours, the microhardness of each specimen was measured using a Vickers indenter, with a load of 100 gm and dwell time of 15 seconds (HV 0.2/40).

The specimens were further subjected to VH test in an interval of 7, 30, and 90 days. The data were subjected to statistical analysis—Student’s t test, analysis of variance, and *post hoc* Tukey’s test.

**Results:**

The present study showed that SDR™ in bulk curing showed consistently greater value of hardness and was comparable to traditional incremental cured Filtek Z350, highlighting the advantages of the new SDR technology.

**How to cite this article:** Sarma A, Nagar P. A Comparative Evaluation of Time-dependent Changes on the Surface Hardness of Bulk Cure Composites: An *in vitro* Study. Int J Clin Pediatr Dent 2018;11(3):183-187.

## INTRODUCTION

Pediatric restorative dentistry is a dynamic combination of ever-improving materials and reliable techniques. It is often challenging for a pediatric dentist to deliver a quality restoration in a pediatric patient as behavioral cooperation varies in children.

Hence, faster and easier methods of restoration and restorative materials have to be evaluated from time to time. The use of light-activated composite resins has been increasing day by day considerably due to better esthetics and strong restorations.^1^ The bonding ability of resin-based composite (RBC) to tooth structure lessens the need for mechanical retention in the cavity preparation, thus reducing the chair time and it is a desired factor in pediatric restorative dentistry.

Components of dental resin composites are matrix: a plastic resin material that forms a continuous phase and combines the filler particles [bisphenol A glycidyl meth-acrylate (bis-GMA), urethane dimethacrylate (UDMA), triethylene glycol dimethacrylate (TEGDMA)]; fillers: Reinforcing particles and (or) fibers that are dispersed in the matrix (silica, fused silica); coupling agent: Bonding agent that promotes adhesion between filler and resin matrix, i.e., methacryloxypropyltrimethoxy silane.

Variations in this basic chemistry have produced a range of composites with distinct properties and different handling characteristics.^2^ Since the development of resin composite restorative material, there has been outstanding improvement of this material. The development has been more focused on the quantity of the filler and polymerization.

In the polymerization of RBC, shrinkage occurs due to the change from carbon single to double bonds. This event, called polymerization shrinkage, causes stress on the cavity walls and separates the composite material from cavity walls. Surface hardness is a well-accepted indicator of the polymerization degree and has been used in many studies.^1^

There are different photoinitiators for starting polymerization in composites. Traditionally, composite materials are required to be cured in increments leading to increased chair time which is a disadvantage in pedi-atric dentistry. Nevertheless, it is an innovative idea of a self-adapting material as bulk, which is time saving and easy to manipulate in the working time.^3^

Polymerization of light-activated composite resins starts at the surface, where light is applied^2^ due to which the deepest parts are abstained from complete polymerization leading to discoloration, secondary caries and sensitivities of the teeth.^4^

The degree of polymerization of resin-based restorative materials can be analyzed directly or indirectly using different techniques. There are direct methods like laser Raman spectroscopy and infrared spectroscopy, but are complex, costly, and time consuming. Indirect methods include scraping, visual evaluation, and surface hardness tests.^1^

In recent times, many researches have been conducted in RBC technology, improving the chemical and physical properties of composite, e.g., new monomers, translu-cency, initiator systems, and filler technology. Two new bulk cure materials, Tetric N Ceram and SDR™, have been introduced which claim to be nano-optimized 4 mm composites, which were evaluated in this study.

## MATERIALS AND METHODS

This *in vitro* study was done to evaluate the surface hardness through VH test of one conventional hybrid resin composite, compared with that of two bulk cure resin composites. This study was carried out in the Department of Pedodontics and Preventive dentistry in Krishnadevaraya College of Dental Sciences, Bengaluru, Karnataka, India.

## ARMAMENTARIUM

- Aluminum mold
- One hybrid composite; Filtek Z350—3M ESPE
- One packable bulk fill composite; Tetric N Ceram bulk fill—Ivoclar
- One flowable bulk fill composite; SDR™—DENTSPLY/ Caulk
- VH Tester—Model-NEXUS EW4304, Bower’s Metrology, ESEWAY, UK
- Deionized water
- Transparent plastic matrix strip
- Composite light curing gun
- Ball burnisher

### A. Filtek Z350 (3M ESPE)

The resin system is slightly modified from the original Filtek™ Z250 Universal Restorative and Filtek™ Supreme Universal Restorative resin. Resin: Bis-GMA, UDMA, TEGDMA, and bis-ethoxylated dimethacrylate (EMA)^6^ resins; fillers: Combination of non-agglomerated/ non-aggregated 20 nm silica filler, non-agglomerated/ non-aggregated 4 to 11 nm zirconia filler, and aggregated zirconia/silica cluster filler (comprised of 20 nm silica and 4-11 nm zirconia particles).

Filler size: 20 nm silica and 4 to 11 nm zirconia; Filler volume: 55.6%; Filler weight: 72.5%

### B. The SDR™ (DENTSPLY) Resin

Ethoxylated bisphenol A dimethacrylate (EBPADMA); TEGDMA; modified UDMA resin; Filler: Barium-alumino-fluoro-borosilicate glass; Strontium alumino-fluoro-silicate glass; Filler volume: 44% Filler weight: 68% Photoinitiator: Camphorquinone (CQ); Diulents: Butylated hydroxyl toluene (BHT); UV Stabilizer Filler;

### C. Tetric N Ceram Bulk fill Resin

Dimethacrylates: Bis-GMA, bis-EMA and UDMA.

Filler: (barium aluminum silicate glass with two different mean particle sizes, an Isofiller, ytterbium fluoride and spherical mixed oxide) in order to achieve the desired composite properties. Filler size: 0.4 to 0.7 m Filler volume: 61% Photoinitiators: Camphorquinone and acyl phos-phine oxide together with a recently patented initiator ivocerin-dibenzoyl germanium derivative. Table 1 shows comparative properties of the materials used in the study and Flow chart 1 shows the sample distribution.

**Table 1:** **Table 1:** Comparative properties of the resins

*Material*		*Content*		*Filler content*		*Filler particle size*		*Photoinitiator*	
Z350 (3M ESPE)		**Resin:** Bis-GMA, UDMA, TEGDMA, and bis-EMA (6) resins **Fillers:** Combination of non-agglomerated/non-aggregated 20 nm silica filler, non-agglomerated/non-aggregated zirconia filler, and aggregated zirconia/silica cluster filler		Volume: 55.6% Weight: 72.5%		20 nm silica and 4-11 nm zirconia		CQ	
SDR™ (Dentsply)		**Resin:** EBPADMA; TEGDMA; modified UDMA resin **Filler:** Barium-alumino-fluoro-borosilicate glass; Strontium alumino-fluoro-silicate glass		Volume: 44% Weight: 68%				CQ	
Tetric N Ceram bulk fill (Ivoclar)		**Resin:** Dimethacrylates: Bis-GMA, Bis-EMA, and UDMA **Filler**: Barium aluminum silicate glass with two different mean particle sizes, an Isofiller, ytterbium fluoride and spherical mixed oxide		Volume: 61%				CQ and acyl phosphine oxide together with a recently patented initiator Ivocerin-dibenzoyl germanium derivative	

**Flow Chart 1:**
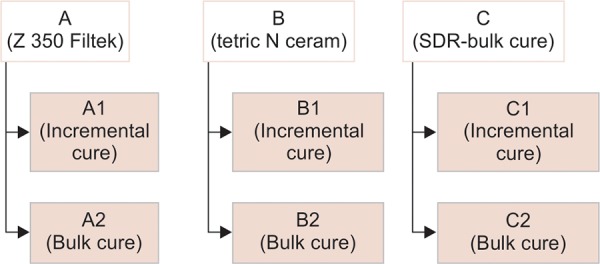
Sample distribution

## DATA COLLECTION

Sample size—60 specimen preparation and VH: 20 specimens of each material were prepared in cylindrical aluminum molds with an internal diameter of 5 mm and depth of 4 mm, 10 specimens in incremental curing (subgroups I1, II1 and III1) and 10 specimens in bulk curing (subgroups I2, II2, and III2).

The surface of each specimen was covered with a transparent plastic matrix strip before light curing with conventional visible light for 40 seconds. This was done to avoid formation of oxygen-inhibited superficial layer, which is known to have a lower hardness. The specimens were then detached from the molds and finished with a fine sand paper.

For groups I and II, the condensable material was dispensed directly on to the mold slot and manipulated using a ball burnisher to assure that there was no gap between the slot walls and the material. For group III, the flowable material was dispensed from the compula tips using a gun provided by the manufacturer. For incremental curing, material was placed till 2 mm and cured, followed by placement of another 2 mm of material and again cured.

For bulk curing, the entire slot of 4 mm depth was filled with material and was cured. The specimens thus obtained were then stored in deionized water and transferred to an incubator at 37°C for 24 hours to simulate clinical conditions. After 24 hours, the microhardness measurement of each specimen was recorded using a Vickers indenter, with a load of 100 gm and dwell time of 15 seconds (HV 0.2/40).

The specimens were further subjected to VH test at intervals of 7, 30, and 90 days. FORMULA:

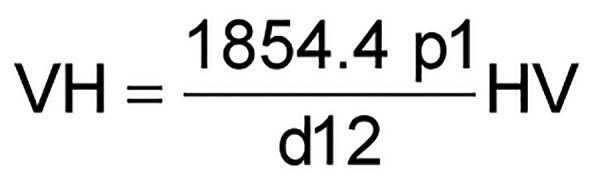



where, p1 = load in gram force, d1 = mean diagonal of indentation in mm.

## RESULTS

**Graph 1:**
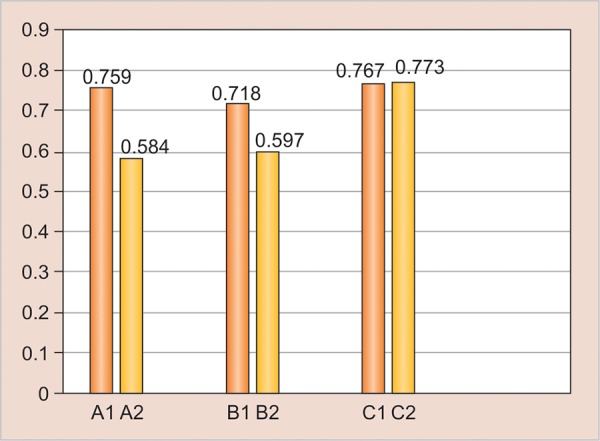
Result—24 hours

Graph 1 (results after 24 hours) reveals that after 24 hours, there was a significant difference in the hardness of I1 and II2, but there was no significant difference in hardness between the other groups.

**Graph 2:**
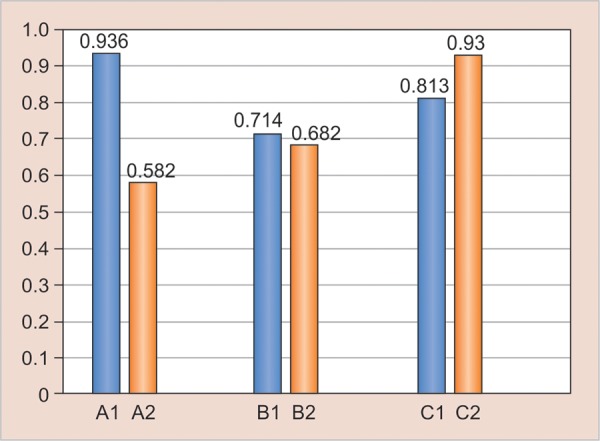
Result—7 days

It showed that Filtek Z350 showed more hardness in incremental curing than bulk curing while other two materials showed comparable hardness in both incremental as well as bulk curing. Graph 2 (results after 7 days) shows that after 7 days, I1, I2, and III2 showed significant difference, but there was no significant difference seen between II1 and II2.

**Graph 3:**
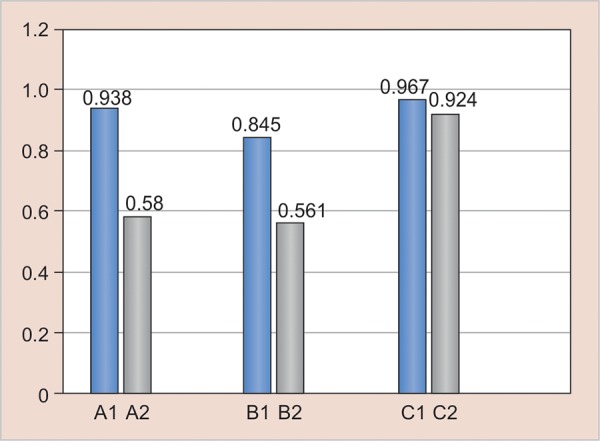
Result—30 days

**Graph 4:**
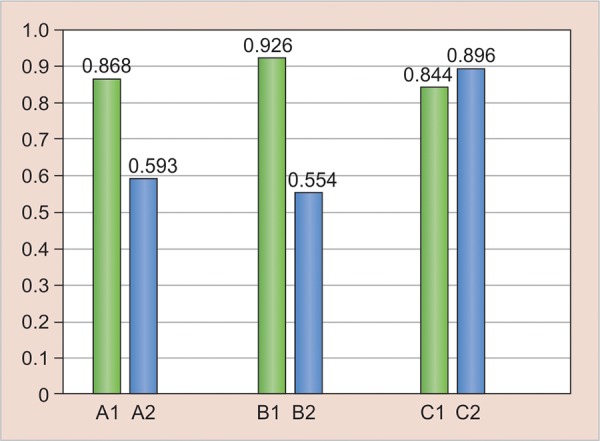
Result—90 days

In 1 week’s time, the hardness of Filtek Z350 and SDR increased, but there was no significant increase in the hardness of Tetric N Ceram. Graph 3 (results after 30 days) shows that after 30 days, all the groups showed significant difference in hardness. All the specimens exhibited an increase in hardness due to continued polymerization reaction. Graph 4 (results after 90 days) reveals that after 90 days, except for and III2, all other groups showed significant difference. The SDR groups showed consistent results showing that the polymerization was completed after 30 days itself and there was no monomer left in the specimen. But, however, the other two materials still showed increase in the hardness due to further degree of conversion.

Intergroup comparison between the incremental and bulk cure groups of each material showed the variation within the same material. After 24 hours, SDR in bulk curing showed the highest hardness followed by SDR in bulk curing > Filtek Z350 in incremental curing > SDR in incremental curing > Tetric N Ceram in incremental curing > Tetric N Ceram in bulk curing > Filtek Z350 in bulk curing.

After 7 days, both Filtek Z350 in incremental curing and SDR in bulk curing had highest and equivalent hardness (0.93) followed by SDR in incremental curing > Tetric N Ceram in incremental curing > Tetric N Ceram in bulk curing > Filtek Z350 in bulk curing. After 30 days, there was a slight variation in the hardness of the specimens.

The SDR in incremental curing > FiltekZ350 in incremental curing > SDR in bulk curing > Tetric N Ceram in incremental curing > Filtek Z350 in bulk curing > Tetric N Ceram in bulk curing. After 90 days, there was significant increase in the hardness exhibited by Tetric N Ceram followed by SDR in bulk curing > Filtek Z350 in incremental curing > SDR in incremental curing > Filtek Z350 in bulk curing > Tetric N Ceram in bulk curing.

## DISCUSSION

The present study has shown that microhardness values of composite resins are not constant but increase with time which is in accordance with the study done by Ozcan et al.^1^ It is also in accordance with the results of the study conducted by Alshali et al^5^ where microhardness of immediately postcured bulk fill composites and 24 hours postcured bulk fill composite in dry storage composite showed a significant difference.

A continuous increase of VH up to 1 week has been observed with about 92% of the maximum hardness achieved at 24 hours and slightly higher VH hardness values at 37°C compared with 23°C storage temperature. The increase in percentage microhardness after 24 hours of dry storage in the current study is mainly attributed to progressive cross-linking reaction and post-irradiation polymerization; hence, it can be used as an indirect measure to assess changes in the degree of conversion of the resin matrix.^5^

Researchers suppose that unreacted free radicals in the structure lead this event by continuing to generate crosslinks after light application.^1^ The reason why packable bulk cured specimens showed less hardness than flow-able composite can be due to inadequate light reaching the deep parts of the composite material.^6^

This result of our study is in accordance with the study done by Li et al^7^ where they have found that the flow-able bulk fill RBCs showed a higher “effective” curing area than the fiber-reinforced RBC, revealing a higher “effective” curing area than the bulk fill and conventional (control) RBC.

Only the flowable bulk fill RBCs were able to be cured “effectively” at a 4-mm depth for the complete specimen dimension (up to 4 mm outside the light beam). The polymerization efficiency at greater depth of SDR (Dentsply) should probably be ascribed primarily to its high translucency, allowing more transmission of light through the material.

Our measurement of transmitted light irradiance showed that up to a depth of 4 mm, it was higher for SDR (Dentsply) than for the other RBCs tested, except for Filtek bulk fill flowable (3M ESPE).^7^ Our study also showed that packable composite Tetric N Ceram and conventional nano hybrid composite Filtek Z350 showed better micro-hardness in incremental curing than bulk curing which is in accordance with the study done by Abed et al.^8^

Tetric N Ceram showed good result in incremental curing, but failed to show adequate hardness in bulk curing. The main reason must be inadequate polymerization due to insufficient depth of cure. Many composites with low viscosity were reported to be optimally polymerized up to a depth of 4 mm.^7^

The overall impression of the result of our study shows that flowable composite with SDR technology can be used in bulk cure up to 4 mm without compromising the hardness of the restoration. Our main aim was to find out comparability of the bulk cure composite with that of traditional methacrylate-based Filtek Z350. The SDR™ showed comparable hardness to conventional nanohybrid Filtek Z350, which is not in accordance with the study conducted by Ilie and Hickel.^9^

The effectiveness of the photopolymerization process can be measured by the degree of conversion (i.e., percentage of the reacted aliphatic carbon-carbon double bonds) and has been directly correlated with mechanical properties (e.g., hardness and shrinkage) of the composite resin-based materials.

Unreacted monomers and/or functional groups within the polymer can act as plasticizers and therefore, have a negative impact on the mechanical properties.^10^ The main reason for the increased hardness of SDR composite must be attributed to its patented “stress decreasing resin,” which does not reduce the filler content as much as the other flowable composite.

It retains the flowable properties and the translucency of the material allows more depth of cure, leading to complete polymerization of the material and hardness. It was claimed that resin systems based on the SDR™ technology with a polymerization modulator being chemically embedded in the polymerizable resin backbone controls polymerization kinetics which will induce lower polymerization shrinkage in the flowable composite based on it.^7^

## CONCLUSION

From the results of the present study, it can be concluded that:

- SDR™ in bulk curing showed consistently greater value of hardness and was comparable to traditional incremental cured Filtek Z350.
- Tetric N Ceram® showed least hardness value in bulk curing.
- Hence, it was concluded that SDR™ can be placed in bulk up to 4 mm in class I and II cavities without compromising on the hardness and retaining the flow-able properties.

This may result in time savings up to 40% as compared with the laborious conventional incremental technique. Within the limitations of this study, bulk cure composites appear to be a boon for pediatric dentistry by reduced chair time and increased durability.

However, more clinical researches are required in this field.
